# Hospitalisations for respiratory tract infections in preterm and term born children aged 1–5 years before, during and after COVID-19 control measures: an interrupted time-series analysis

**DOI:** 10.1136/bmjpo-2025-004270

**Published:** 2026-09-01

**Authors:** Andreas Asheim, Kari Risnes, Isobel Todd, Henrik Døllner, Eero Kajantie, Signe Opdahl, Sara Marie Nilsen

**Affiliations:** 1 Center for Service Innovation, Trondheim University Hospital St Olav’s Hospital, Trondheim, Norway; 2 Department of Clinical and Molecular Medicine, Faculty of Medicine and Health Sciences, Norwegian University of Science and Technology, Trondheim, Norway; 3 Children’s Clinic, Trondheim University Hospital St Olav’s Hospital, Trondheim, Norway; 4 Department of Paediatrics, The University of Melbourne, Parkville Melbourne, Victoria, Australia; 5 Murdoch Children’s Research Institute, Parkville Melbourne, Victoria, Australia; 6 Research Unit of Clinical Medicine, Medical Research Center Oulu, University of Oulu and Oulu University Hospital, Oulu, Finland; 7 Population Health Unit, Finnish Institute for Health and Welfare, Oulu, Finland; 8 Department of Public Health and Nursing, Norwegian University of Science and Technology, Trondheim, Norway

**Keywords:** COVID-19, Epidemiology, Health Policy

## Abstract

**Background:**

Preterm-born children are vulnerable to respiratory tract infections (RTIs), but we know little about how these were affected by COVID-19 pandemic control measures.

**Methods:**

We studied Norwegian population-wide registry data on 1–5-year olds born 2012–2021. RTI hospitalisations were identified from primary hospitalisation diagnosis codes. We modelled prepandemic hospitalisation rates, January 2017 to March 2020, using quasi-Poisson regression with harmonic seasonal terms. These were compared with monthly hospitalisation rates, March 2020 to December 2022. The calculations were performed separately for gestational age groups: 23–33, 34–36, 37–41 completed weeks.

**Results:**

We identified 9734 RTI hospitalisations among 449 105 children aged 1–5 years, with higher rates in the most preterm groups. Across gestational ages, we observed lower hospitalisation rates during control measures in 2020, followed by a surge after measures were lifted in 2021. For children born in weeks 23–33, the rate reduction with 95% CI during the first year of the pandemic compared with the prepandemic years was −21 per 1000 person-years (95% CI −30 to −13), whereas in April–December 2022, there was an increase of 22 (95% CI 12 to 31). For term born (week 37–41), the corresponding changes were −4.9 (95% CI −5.5 to −4.3) and 3.6 (95% CI 2.9 to 4.2).

**Conclusions:**

Compared with term born, preterm-born children of all gestational age groups were more distinctly protected by pandemic control measures. They were also more affected by the resurgence of infections when measures were lifted. These findings call for policies to protect preterm-born children better from RTIs, especially when viral spread is high.

**WHAT IS ALREADY KNOWN ON THIS TOPIC**

- In periods with COVID-19 pandemic control measures, paediatric infection-related hospitalisation rates increased, and there were surges in infections after they were lifted.
- Children born preterm are vulnerable to severe respiratory tract infections.

**WHAT THIS STUDY ADDS**

- We show that the lowest gestational age groups were particularly impacted by the high spread of respiratory pathogens post-COVID, but that impact in late preterm born was also at substantially higher risk than term-born children.

**HOW THIS STUDY MIGHT AFFECT RESEARCH PRACTICE OR POLICY**

- The study may inform better policies to protect a wide group of preterm-born children from respiratory tract infections, especially when viral spread is high.

## Background

 During the COVID-19 pandemic, control measures were implemented to mitigate the spread of the virus, reduce the incidence of COVID-19 infections and thus maintain capacity in the healthcare services to treat the most severe cases. These measures included societal restrictions like closure of institutions and social distancing, and individuals were encouraged to wear medical face masks, vaccinate and adopt rigorous hygiene practices.^1^ Paediatric infection-related hospitalisations were affected by these measures, with population studies from France, the UK and Australia demonstrating clear and similar patterns: There were sharp decreases in community-acquired infections^2^ and infection-related hospitalisations^3 4^ from the prepandemic to the pandemic period. As control measures were lifted, surges in incidence of paediatric infections were observed, often with out-of-season outbreaks,^5 6^ attributed in part to a population immunity debt, that is, increased susceptibility due to reduced previous exposure with less activation of the immune responses, combined with disruption in vaccine coverage.^7^

A recent analysis of Norwegian data shows that preterm-born children are at high risk of severe respiratory tract infections.^8^ Using data up to 2017, the study showed that children born late preterm (34–36 weeks completed gestation) had about two times the risk of hospitalisation for lower RTIs at ages 1–5 years, compared with term-born peers, while extremely preterm-born children (<28 weeks gestation) faced approximately 10 times higher risk. The reduction in admissions for respiratory infections observed for children overall during periods of COVID-19 control measures likely extends to extremely preterm-born children.^3^ However, their generally higher risk of RTIs may also have made them more vulnerable during the post-pandemic resurge in respiratory viruses. Hence, the balance between potential benefits and harms of temporary, widespread control measures on risk of respiratory infections for children-born preterm remains unknown. Such knowledge could help tailor preventive strategies for this vulnerable subpopulation, both during future pandemics and seasonal outbreaks of common RTIs. Our primary aim was to assess whether patterns of childhood hospitalisations with RTIs before, during and after the COVID-19 pandemic differed according to gestational age at birth. Second, we aimed to estimate cumulative differences in RTI hospitalisations through the pandemic and postpandemic period.

## Methods

In this population-based analysis, we used data from the Medical Birth Registry of Norway (MBRN).^9 10^ A unique and anonymous identification number derived from the national identity number enabled us to link patient information across multiple registries. Records on all live births from 2012 to 2021 were linked to data from the Norwegian Patient Registry (NPR) on all admissions with RTIs from 2017 through to 2022. Data from NPR are routinely analysed by the National Service for Validation and Completeness Analyses and considered to have a high level of completeness.^11^ Diagnoses are assigned at discharge by a specialist. Dates of death were available from the Norwegian Cause of Death Registry,^12^ while dates of emigration were available from Statistics Norway. The study and data linkage were approved by the Regional Committee for Medical and Health Research (REK-Midt 2018/32) and the registry-keeping authorities.

Individuals were excluded if their identification number or birth weight was missing, if gestational age at birth was below the threshold for active neonatal treatment (<23 weeks) or post-term (>41 weeks). To reduce the impact of incorrect measures of exposure, we excluded individuals with birthweights>6000 g or <350 g, birthweights for gestational age >4 or <−6 SD from expected, or if maternal age was >55 years. A flowchart including the number of excluded at each stage is provided in online supplemental figure 1.

Data on gestational age at birth were obtained from the MBRN and estimated based on ultrasound examination, or the last menstrual period if ultrasound was not performed. In the case of assisted reproduction, estimation from date of embryo transfer was preferred over ultrasound. Gestational age in days was converted to completed weeks and categorised as extremely to moderately preterm (23–33), late preterm (34–36), early to full term (37–41). The grouping of extreme (<28 weeks), very (28–31), and moderately (32–33) preterm into one group was presented as the main analysis, while rates for each WHO category were presented in supplementary analysis.

The primary outcome was hospitalisation with a main diagnosis of RTI between ages 1 and 5 years. *We excluded hospitalisations during the first year after birth where time at risk was not precise in the data, particularly for preterm groups needing extensive newborn hospital care and follow up*. NPR collects data on diagnoses based on the International Classification of Diseases (ICD-10) for administrative purposes, and we considered all the following codes as indication of RTI: J00-J06, J09-J18, J20-22, J32, J39.0, J39.1, J85 and J86. These include bacterial and viral upper and lower respiratory tract infections (LRTI). In supplementary analyses, we consider the codes J20-22 as indicating LRTI, and J13-J15, J16.0, J17.0, J20.0-2 as indicating a bacterial RTI. From 2020, the code U07 was used to indicate admission for COVID-19, in combination with a pneumonia code. See online supplemental table 1 for specification of these codes.

### Statistics

Monthly numbers of hospitalisations and person-time at risk were calculated for each gestational age group. We used data from January 2017 to March 2020 to model prepandemic rates of hospitalisation, using quasi-Poisson regression with four harmonic terms to model seasonal variation and an offset parameter to account for person-time at risk. Details on the implementation of this method including a sensitivity analysis addressing modelling of seasonality is provided as a methods supplement in the online supplemental materials, methods supplement. This model was extrapolated to provide expected rates of admissions based on information from prepandemic years to compare with observed rates during the pandemic (April 2020—March 2022) and postpandemic (April 2022—December 2022). We computed monthly differences in admission rates and cumulative differences in the number of hospital admissions. Estimates with 95% CIs were computed using 10 000 resamples to compute bootstrapped SEs.

### Additional analyses

To assess the impact of aggregating all extremely to moderately preterm-born into one group, we calculated admission rates for each gestational age group to compare with the aggregated rates. We did an additional analysis restricted to hospitalisations due to LRTIs to investigate a narrower definition of the outcome.

## Results

In total, we identified 6983 hospitalisations with RTIs from 2017 to 2022 among 265 055 children. After March 2020, there were 4535 RTI hospitalisations, among which 444 were coded with an ICD-10-code indicating COVID-19. The overall prepandemic rates of RTI hospitalisations, per 1000 person-years, were 34 for the 23–33 weeks group, 15 for the 34–36 weeks group and 7 for term born (table 1).

**Table 1:** Key study population statistics

	Extremely to moderately preterm (23–33 weeks)	Late preterm (34–36 weeks)	Early to full-term (37–41 weeks)
Number of individuals	4285	12 039	248 731
Person-years follow-up time,	10 547	29 573	606 229
Hospital admissions, n (rate per 1000 py)	2124 (155)	3275 (84)	37 987 (48)
For RTI, n (rate per 1000 py)	465 (43)	558 (18)	5930 (9)
For LRTI, n (rate per 1000 py)	282 (26)	254 (8)	2361 (4)
For bacterial RTI, n (rate per 1000 py)	29 (3)	48 (2)	586 (1)
Females, n (%)	2340 (54)	6404 (53)	126 502 (51)
Mothers’ age at birth (years), median (IQR)	31 (8)	31 (7)	31 (7)

LRTI, lower RTI; py, person-years; RTI, respiratory tract infection.

**Figure 1:**
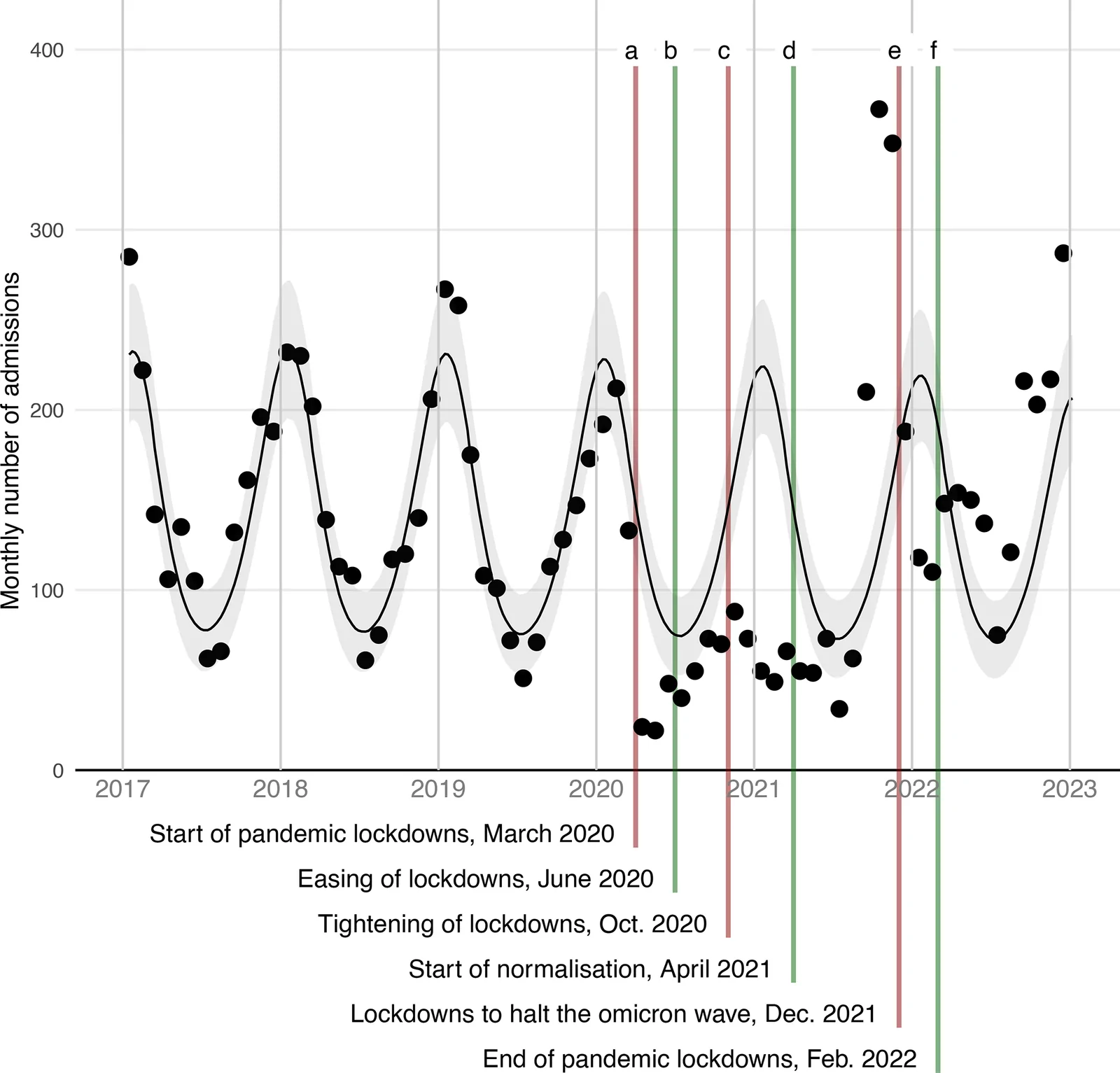
Monthly number of hospitalisations due to respiratory tract infections in children aged 1–5 years from 2017 to 2022. The predicted numbers of admission based on pre-pandemic admissions, using quasi-Poisson regression with harmonic seasonal terms, are shown as a continuous curve with a 95% confidence band. Red lines indicate the onset of severe pandemic restrictions, green lines indicate lifting of restrictions.^40^ (a) Start of pandemic lockdowns, March 2020. (b) First easing of lockdowns, June 2020. (c) Tightening of lockdowns, October 2020. (d) Start of normalisation, April 2021. (e) Omicron lockdown, December 2021. (f) End of lockdowns, February 2022.

Figure 1 shows the monthly number of admissions for RTIs in the study period, with numbers in online supplemental table 2. After initiation of extensive preventive measures from March 2020, every month from April 2020 to May 2021 had fewer hospitalisations than any of the corresponding previous months. Easing of control measures from April to September in 2021 was followed by a sharp out of season rise in hospitalisations from September to November 2021. In 2022, there was again an earlier and higher peak than during prepandemic years, occurring in October–December.

**Figure 2:**
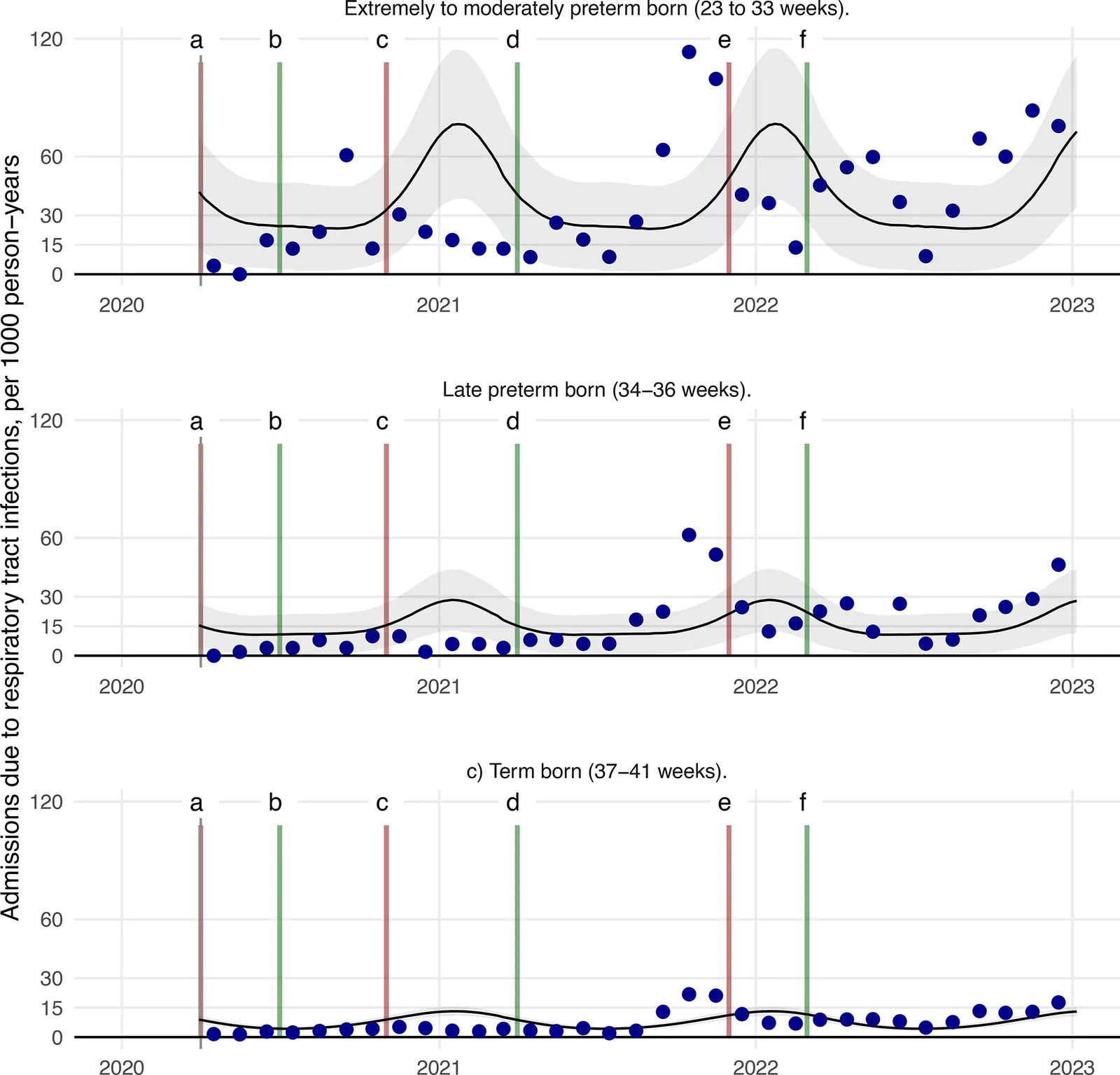
Rates of hospitalisations due to respiratory tract infections during and after the pandemic in children aged 1–5 years. The predicted rates of admission based on prepandemic admissions, calculated with quasi-Poisson regression with harmonic seasonal terms, are shown as a continuous curve with a 95% confidence band. (**a**) Start of pandemic lockdowns, March 2020. (**b**) First easing of lockdowns, June 2020. (**c**) Tightening of lockdowns, October 2020. (**d**) Start of normalisation, April 2021. (**e**) Omicron lockdown, December 2021. (**f**) End of lockdowns, February 2022.

Rates of RTI hospitalisations during and after the pandemic are shown by gestational age categories in figure 2. There was a similar seasonal pattern of hospitalisations across the gestational categories in this period, with consistently low rates from April 2020 to August 2021. In October 2021 there was a peak with higher rates than at any point since 2017 when admissions for RTI per 1000 person-years were 113 for the 23–33 weeks group, 62 for the 34–36 weeks group and 22 for term born (online supplemental table 3). Considering the extremely, very and moderately preterm born separately in an additional analysis (online supplemental figure 2) indicates that rates were substantially higher by lower gestational age and highest for the extremely preterm born.

During the first pandemic year (April 2020 to March 2021), the hospitalisation rate for the extremely to moderately preterm-born group (23–33 weeks) was 21 lower per 1000 person-years (95% CI 13 to 30) than before the pandemic. For the gestational age groups 34–36 and 37–41 weeks, the reductions were 11 (95% CI 7.7 to 15) and 4.9 (95% CI 4.3 to 5.5), respectively. The postpandemic period (April 2022 to December 2022) had corresponding increases in rates of hospitalisations for each gestational age group, mirroring the reductions during the first pandemic year (table 2). When considering a narrower outcome definition only counting LRTI admissions, the results were similar, with a substantial reduction in hospitalisations for the most preterm born in the start of the pandemic (31 fewer per 1000 person-years, 95% CI 19 to 46), see online supplemental table 4.

**Table 2:** Observed versus predicted rates of RTI admissions, per pandemic year, among children aged 1–5 years born in Norway 2012–2021, according to gestational age at birth

		Extremely to moderately preterm (23–33 weeks)	Late preterm (34–36 weeks)	Early to full-term (37–41 weeks)
April 2020 to March 2021				
	Rate per 1000 person-years	19	5.2	3.3
	Difference vs predicted rates	−21 (−30 to −13)	−11 (−15 to −7.7)	−4.9 (-5.5 to −4.3)
Apr 2021 to March 2022				
	Rate per 1000 person-years	41	21	8.9
	Difference vs predicted rates	1.5 (−7.5 to 10)	5.1 (1.2 to 8.7)	0.7 (0.0 to 1.3)
April 2022 to December 2022				
	Rate per 1000 person-years	53	22	10.5
	Difference vs predicted rates	22 (12 to 31)	8.5 (4.1 to 12)	3.6 (2.9 to 4.2)

Predicted rates of admission based on pre-pandemic admissions, calculated with quasi-Poisson regression with harmonic seasonal terms. Bootstrapped 95% CIs.

RTI, respiratory tract infection.

**Figure 3:**
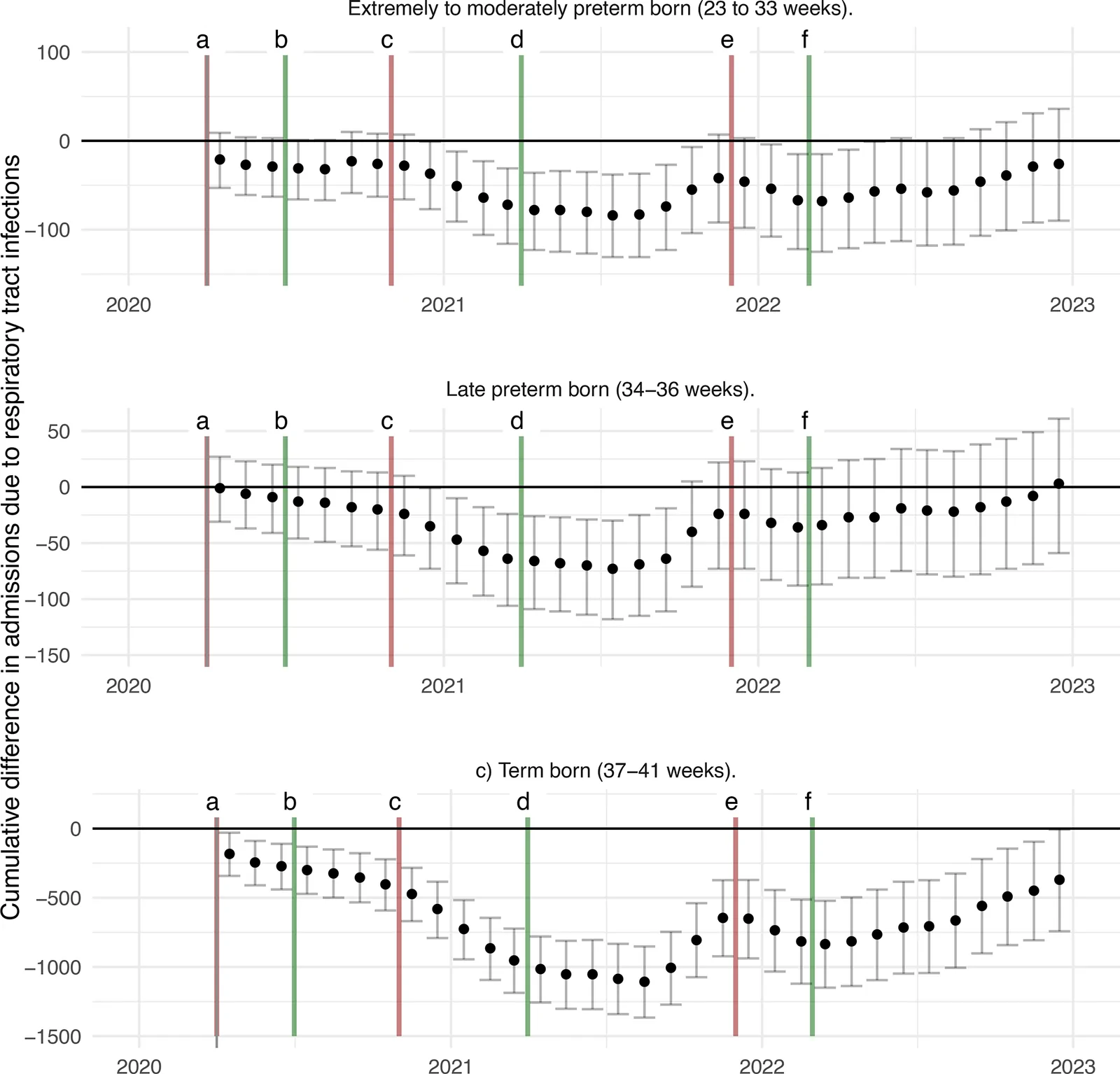
Monthly cumulative difference between the predicted and actual number of admissions due to respiratory tract infections in children aged 1–5 years, born in Norway 2012–2021. The predicted number of admissions was based on pre-pandemic admissions (January 2017– March 2020), calculated with quasi-Poisson regression with harmonic seasonal terms. Monthly estimates presented with a 95% bootstrapped CI. (**a**) Start of pandemic lockdowns, March 2020. (**b**) First easing of lockdowns, June 2020. (**c**) Tightening of lockdowns, October 2020. (**d**) Start of normalisation, April 2021. (**e**) Omicron lockdown, December 2021. (**f**) End of lockdowns, February 2022.

Figure 3 shows the cumulative difference between RTI admissions and predicted admission (numbers in online supplemental table 5). By December 2022, the cumulative number of observed admissions was close to the expected number for the preterm-born groups, with differences of −26 (95% CI −90 to 36) for week 23–33 and 3 (95% CI −59 to 61) for week 34–36. For term born, the cumulative difference by the end of 2022 was still negative −370 (95% CI −742 to −7).

## Discussion

Across gestational categories, rates of RTI hospitalisations decreased substantially during periods of extensive COVID-19 control measures, followed by a sharp rise when measures were eased. The most pronounced changes in absolute rates were among children born extremely to moderately preterm (23–33 weeks), who also had substantially higher prepandemic admission rates. The decline in admissions at the start of the pandemic was largely offset by the resurgence in admissions after easing control measures.

### Reductions in paediatric infections during COVID-19 pandemic

Hospitalisations for childhood infections were significantly reduced during the COVID-19 pandemic in countries that applied behavioural and societal preventive strategies. Estimates from time-series analysis on population data from Australia showed reductions were most marked in children under 5 years and for LRTIs.^4^ Similar effects have now been extensively documented in several countries including the UK,^3^ Denmark^13^ and the Netherlands,^14^ indicating around an 80% reduction in hospitalisations for bronchiolitis and influenza.^3^ A multicentre analysis^14^ reviewed and evaluated temporal changes in paediatric hospitalisations (not limited to infections) during the COVID-19 pandemic period compared with pre-COVID periods and concluded that the overall reduction in hospital admissions was 40%–70%.^3^ A recent review of the global burden of respiratory syncytial virus (RSV) hospitalisation in young children during and after the COVID-19 pandemic^14^ concluded that, compared with 2019, the rates of RSV-associated hospitalisations in children aged 0–60 months in 2020 decreased by 80% in high-income countries and 14% in upper middle-income countries. Among the RTI hospitalisations in our study, RSV was likely a major contributor,^15^ and the marked and rapid reduction in childhood RTI and LRTI hospitalisation observed during the COVID-19 pandemic is, therefore, in line with these studies.

Few studies have assessed effects of the pandemic on hospitalisation in paediatric high-risk groups such as preterm-born children. In the UK database study on paediatric infection admissions,^16^ children born extremely preterm and children with bronchopulmonary dysplasia were studied. Although separate analyses were not performed, reductions in hospitalisation rates were reported to be comparable across groups of co-morbidities.

Among all original studies and supplemental materials included in a recent review on RSV during and after the COVID-19 pandemic,^17^ no studies had estimated effects on RTI hospitalisations in preterm-born children. Some cohort studies have reported proportions of preterm born among hospitalised children before and after the pandemic but can draw no firm conclusions due to small samples. A Spanish centre reported a higher proportion of preterm born among hospitalised children with RSV during the pandemic than before,^18^ while similar comparisons from France^19^ and Croatia^19^ did not support changes in the proportion of preterm born among hospitalised children.

### Surge after lifting of pandemic control measures

After pandemic control measures were lifted in early 2021, substantial resurgences and out-of-season outbreaks in childhood respiratory viral infections, including RSV outbreaks, were observed in many countries, in line with our findings from Norway. A Canadian population-based study^20^ on RSV-associated hospitalisations in children younger than 5 years found that the 2021–2022 season to have comparable rates to those before the pandemic while the 2022–2023 season resulted in two times as many hospitalisations as the prepandemic seasonal average. Later, Canadian data showed that in the 2023–2024 season, patterns of RSV and other RTI hospitalisations approached prepandemic levels.^21^ The global RSV review^17^ concluded that the surge in 2021 was ended and infections had returned to prepandemic level by March 2022.

In an analysis similar to ours, data from a French national hospital database from 2015 to March 2023 were used to estimate changes in lower RTI hospitalisations during and after the pandemic among children <18 years. The results showed a sharp decrease during the pandemic control period followed by a marked increase after preventive measures were lifted. The authors concluded that although the resurgence rates exceeded the pre-pandemic baseline trend, the overall cumulative effects on hospitalisation in children were still beneficial at the end of the study period but did not present results specifically for preterm born or other high-risk groups.^22^

The peak in hospital admissions after lifting control measures in 2021 may indicate a shift in the timing of infections, with older children now experiencing respiratory infections from microorganisms they would usually have encountered at a younger age. Postpandemic outbreaks of childhood infections included not only common respiratory viruses but also invasive bacterial infections such as invasive group A Streptococcal (GAS) disease. Findings in a recent large European analysis suggest the increase in iGAS pneumonia was associated with RSV and influenza activity.^23^ Studies on RTIs after restrictions were lifted showed that preschool children had a gap in humoral immunity with lower levels of protecting antibodies against, for example, influenza-virus and RSV compared with earlier years.^24 25^ However, evidence on immunological responses to infections in preterm-born children is scarce, and it is not known if a similar gap in humoral immunity has occurred across all gestational ages.

### *S*trengths and limitations

A key strength of this study is the use of nationwide registry data, which ensures near-complete population coverage. The Norwegian healthcare system provides free access to public healthcare for all children under 16 years, which limits the influence of socioeconomic factors on hospitalisation rates. The inclusion of data from the postpandemic period allows for timely analysis of postpandemic trends, although the follow-up time remains limited.

The study also has limitations. Seasonal variation in specific infection types, such as RSV, was not examined in detail, and only hospital admissions were analysed, without information on duration of stay, severity or treatment. There could also have been a higher threshold for admission during periods of pandemic control measures, which could result in the less severe cases not being hospitalised. These factors may lead to an underestimation of disease burden during times of pandemic control measures. During outbreaks of respiratory viruses in late 2021 and 2022, limited capacity at hospitals to receive infected children may also have led to underestimation of disease burden.

Norway implemented a range of non-pharmaceutical interventions to limit the spread of COVID-19 during 2020–2021, including periods of school and kindergarten closures, work-from-home recommendations, restrictions on social gatherings, reduced travel and general guidance on physical distancing, while access to outdoor activities was largely maintained.^26^ Stringency and duration of measures varied substantially across European countries.^27^ The relatively shorter and less continuous restrictions in Norway, together with cross-country differences in compliance and context, may limit generalisability of our findings.

The analysis included individuals born during a time span of 10 years, when the distribution of the youngest gestational age group may have changed marginally due to increased survival. Also, in 2021, during resurge, the children in the 1–5-year group would have different immunity since the youngest were born during the pandemic with altered transplacental antibody transfer, reduced infant microbial exposure and (potentially) disrupted vaccination schedules. When using a repeated cross-sectional design, results could have been affected by such differences between birth cohorts and their histories at different times of the study.

### Interpretation

The present findings support that preventive measures applied during the pandemic were effective in reducing RTI-related hospitalisations also for preterm-born children, and the absolute reductions in rates among preterm born were more substantial than among term-born children. Furthermore, our results demonstrate a higher increase in rates of lower RTI admissions in preterm-born children than in term-born children when viral spread was high during the post-COVID-19 resurgence in infections, indicating vulnerability to more severe disease.

Although the highest absolute hospitalisation rates were observed among the rather wide gestational age range of children born extremely to moderately preterm, an additional analysis shows that the extremely preterm born were particularly vulnerable. Nevertheless, the preterm population is numerically dominated by children born late preterm. Even modest relative changes in hospitalisation risk in this group therefore translate into substantial absolute numbers at the population level. The marked reduction in RTI hospitalisation among late preterm children during periods of COVID-19 measures, followed by a clear rebound after measures were lifted, highlights that this large gestational subgroup is highly responsive to changes in community transmission. From a policy perspective, these findings are relevant as children born late preterm are in many cases not included in targeted prevention strategies, including vaccination policies and RSV prevention programmes. The observed sensitivity of this group to changes in infection pressure suggests that preventive measures with broader reach among preterm groups, such as vaccination, RSV immunisation programmes and infection prevention strategies, may have substantial public health impact beyond targeting only the most extremely preterm infants.

Higher RTI-related admissions by shorter gestational age at birth have been assessed in population studies.^8^ The dominating seasonal pathogen in young children hospitalised with RTI is RSV, and preterm born are at high risk for severe RSV infection. An individual participant meta-analysis found that preterm-born infants accounted for 25% of RSV hospitalisations, despite being a much smaller proportion of the population.^28^ Across 20 studies, preterm-born infants hospitalised with RSV had more intensive care unit admissions, longer stays and higher fatality than term born, while emergency department visit rates were similar.^29^

Increased risk of severe RTIs in preterm-born children may be linked to gestational age-related alterations in the microbiome causing alterations in immune system development^30^ and a respiratory system vulnerable to infections due to small airways and immature local mucosal immune defences.^31^ Furthermore, infection protection in preterm-born neonates is hampered by interrupted maternal transplacental antibody transfer, reducing protective effects of maternal vaccinations and immunity when delivery takes place before full term.

### Infection prevention in preterm born

Although preterm-born children are at high risk of severe respiratory tract infections, implementation of preventive measures specifically tailored for this group is limited. Non-pharmacological measures to prevent the transmission of community-acquired respiratory infection demonstrated substantial efficacies in reducing spread during the COVID-19 pandemic. These infection prevention and control (IPC) strategies included hand hygiene, use of personal protective equipment, social distancing, isolation measures and improved ventilation.^32^

Despite representing a large and clearly identifiable group of children with high susceptibility to community-acquired respiratory infections, evidence specific to preterm-born children is scarce. Few guidelines exist for this group, although studies evaluating postdischarge recommendations on infection prevention in preterm born are being planned.^33^

Immunisations are effective preventive measures to reduce the burden of respiratory tract infections in newborn and young children. Children born preterm have a substantially increased risk of vaccine-preventable RTIs that are targeted in childhood immunisation programmes, such as pneumococci, haemophilus influenza, RSV and pertussis. Safety and efficacy of these vaccines for preterm-born children are well documented and there is support for long-term safety of timely vaccinations according to chronological age also for extremely preterm born infants.^34^ Nevertheless, vaccination timing and coverage in preterm infants are typically poorer than for term born,^35^ resulting in potentially avoidable vulnerability.

Since 2023, new and highly effective opportunities to prevent severe RSV infections in early life have emerged, including maternal vaccination during pregnancy with transplacental antibody transfer^36^ and neonatal immunisations with long-acting monoclonal antibodies.^37^ These approaches have demonstrated high effectiveness in reducing RSV-related hospital admissions during infancy.^38^ The introduction of RSV vaccination and long-acting monoclonal antibodies represent a unique policy window to substantially reduce severe RSV-associated disease in early life in infants born preterm, who are least protected by strategies relying on late-pregnancy vaccination alone. Importantly, while some countries have implemented universal RSV immunisation strategies for all infants during the first RSV season, other preventive approaches remain more selective. Children born late preterm, who constitute the largest preterm subgroup, may not be explicitly eligible for protective immunisations. As RSV prevention policies continue to evolve, our findings highlight the relevance of considering late preterm birth and risk-patterns beyond infancy.

Introduction of additional measures for this group may raise concerns for more isolation for children and their parents outweighing the impact on severe respiratory infections. However, repeated severe respiratory infections already impose a substantial and often unpredictable burden, including recurrent absence from daycare, school and work, as well as considerable strain on healthcare services. Protection from acute RTIs in young children may have benefits well beyond the immediate infection episode.^39^ In this context, additional preventive strategies may represent a different type of burden, one that is more predictable, time-limited and targets periods of greatest vulnerability, rather than an overall increase in restriction or isolation.

## Conclusion

Our study demonstrates a higher burden of RTI hospitalisations in children born at lower gestational ages, and that control measures applied during the COVID-19 pandemic greatly reduced this risk in children of all gestational ages. When control measures were eased, the lowest gestational age groups had the highest rise in hospitalisation rates, but the rise in moderate and late preterm groups was also substantial. The experience of pandemic and postpandemic effects can inform policies for protecting this vulnerable group of children. Evidence and recommendations tailored to prevent severe respiratory infections in preterm-born children should be a priority for better preparedness, especially when spread of respiratory pathogens is high such as during epidemics and pandemics. The balance between negative social effects of control measures and positive effects of avoiding infections could be different in young children at high risk of severe disease, such as preterm-born children.

## Supplementary material

10.1136/bmjpo-2025-004270 online supplemental file 1

## Data Availability

Data are available upon reasonable request.

## References

[1] Skjesol I, Ursin G, Tritter J (2024). Learning to live with COVID-19 in Norway: Moving from a pandemic to an endemic state. Health Policy Technol.

[2] Cohen PR, Rybak A, Werner A (2022). Trends in pediatric ambulatory community acquired infections before and during COVID-19 pandemic: A prospective multicentric surveillance study in France. *Lancet Reg Health Eur*.

[3] Kadambari S, Goldacre R, Morris E (2022). Indirect effects of the covid-19 pandemic on childhood infection in England: population based observational study. *BMJ*.

[4] Todd IMF, Miller JE, Rowe SL (2021). Changes in infection-related hospitalizations in children following pandemic restrictions: an interrupted time-series analysis of total population data. Int J Epidemiol.

[5] Jia R, Lu L, Su L (2022). Resurgence of Respiratory Syncytial Virus Infection During COVID-19 Pandemic Among Children in Shanghai, China. Front Microbiol.

[6] Foley DA, Yeoh DK, Minney-Smith CA (2021). The Interseasonal Resurgence of Respiratory Syncytial Virus in Australian Children Following the Reduction of Coronavirus Disease 2019-Related Public Health Measures. Clin Infect Dis.

[7] Nygaard U, Holm M, Rabie H (2024). The pattern of childhood infections during and after the COVID-19 pandemic. Lancet Child Adolesc Health.

[8] Nilsen SM, Valand J, Rogne T (2023). Gestational age at birth and hospitalisations for infections among individuals aged 0-50 years in Norway: a longitudinal, register-based, cohort study. *EClinicalMedicine*.

[9] Public NIo, Health Medical birth registry of Norway. https://www.fhi.no/en/ch/medical-birth-registry-of-norway/.

[10] Laugesen K, Ludvigsson JF, Schmidt M (2021). Nordic Health Registry-Based Research: A Review of Health Care Systems and Key Registries. Clin Epidemiol.

[11] Bakken IJ, Ariansen AMS, Knudsen GP (2020). The Norwegian Patient Registry and the Norwegian Registry for Primary Health Care: Research potential of two nationwide health-care registries. *Scand J Public Health*.

[12] Health NIoP Norwegian cause of death registry. https://www.fhi.no/en/ch/cause-of-death-registry/.

[13] Bodilsen J, Nielsen PB, Søgaard M (2021). Hospital admission and mortality rates for non-covid diseases in Denmark during covid-19 pandemic: nationwide population based cohort study. BMJ.

[14] Kruizinga MD, Peeters D, van Veen M (2021). The impact of lockdown on pediatric ED visits and hospital admissions during the COVID19 pandemic: a multicenter analysis and review of the literature. *Eur J Pediatr*.

[15] Hasegawa K, Mansbach JM, Teach SJ (2014). Multicenter study of viral etiology and relapse in hospitalized children with bronchiolitis. Pediatr Infect Dis J.

[16] Bourdeau M, Vadlamudi NK, Bastien N (2023). Pediatric RSV-Associated Hospitalizations Before and During the COVID-19 Pandemic. JAMA Netw Open.

[17] Cong B, Koç U, Bandeira T (2024). Changes in the global hospitalisation burden of respiratory syncytial virus in young children during the COVID-19 pandemic: a systematic analysis. Lancet Infect Dis.

[18] Bermúdez Barrezueta L, Matías Del Pozo V, López-Casillas P (2022). Variation in the seasonality of the respiratory syncytial virus during the COVID-19 pandemic. *Infection*.

[19] Fourgeaud J, Toubiana J, Chappuy H (2021). Impact of public health measures on the post-COVID-19 respiratory syncytial virus epidemics in France. *Eur J Clin Microbiol Infect Dis*.

[20] Fitzpatrick T, Buchan SA, Mahant S (2024). Pediatric Respiratory Syncytial Virus Hospitalizations, 2017-2023. *JAMA Netw Open*.

[21] Fitzpatrick T, Buchan SA, Mahant S (2025). Pediatric Acute Respiratory Virus Hospitalizations: A Population-Based Cohort Study, 2017-2024. *J Infect Dis*.

[22] Fafi I, Assad Z, Lenglart L (2025). Did the resurgence of childhood lower respiratory infections offset the initial benefit of COVID-19-related non-pharmaceutical interventions in children? A time-series analysis. *BMC Med*.

[23] Lenglart L, Özmen I, Aguilera-Alonso D (2025). Paediatric invasive group A streptococcal infections and associations with viral infections in 15 European countries after lifting non-pharmaceutical interventions against SARS-CoV-2: an interrupted time-series analysis. *Lancet Reg Health Eur*.

[24] Fossum E, Rohringer A, Aune T (2024). Antigenic drift and immunity gap explain reduction in protective responses against influenza A(H1N1)pdm09 and A(H3N2) viruses during the COVID-19 pandemic: a cross-sectional study of human sera collected in 2019, 2021, 2022, and 2023. *Virol J*.

[25] den Hartog G, van Kasteren PB, Schepp RM (2023). Decline of RSV-specific antibodies during the COVID-19 pandemic. Lancet Infect Dis.

[26] Hallberg A, Aakjaer M, Aaltonen K (2025). Epidemiological outcomes and policy implementation in the Nordic countries during the COVID-19 pandemic. *Arch Public Health*.

[27] Fajgenblat M, Molenberghs G, Verbeeck J (2024). Evaluating the direct effect of vaccination and non-pharmaceutical interventions during the COVID-19 pandemic in Europe. *Commun Med (Lond*).

[28] Wang X, Li Y, Shi T (2024). Global disease burden of and risk factors for acute lower respiratory infections caused by respiratory syncytial virus in preterm infants and young children in 2019: a systematic review and meta-analysis of aggregated and individual participant data. Lancet.

[29] Kenmoe S, Kengne-Nde C, Modiyinji AF (2020). Comparison of health care resource utilization among preterm and term infants hospitalized with Human Respiratory Syncytial Virus infections: A systematic review and meta-analysis of retrospective cohort studies. PLoS One.

[30] Erickson I, Tung J, Schwartz DJ (2025). Consequences of host-microbiome interactions in preterm infants. Infect Immun.

[31] Fortmann MI, Dirks J, Goedicke-Fritz S (2022). Immunization of preterm infants: current evidence and future strategies to individualized approaches. *Semin Immunopathol*.

[32] Organization WH (2024). Global report on infection prevention and control 2024. https://www.who.int/publications/i/item/9789240103986.

[33] Carruthers K, Hannis D, Robinson J (2023). Infection prevention and control measures for preterm infants discharged into the community: a scoping review protocol. *Syst Rev*.

[34] Fortmann I, Dammann M-T, Humberg A (2021). Five Year Follow Up of Extremely Low Gestational Age Infants after Timely or Delayed Administration of Routine Vaccinations. Vaccines (Basel).

[35] Angelidou A, Levy O (2020). Vaccination of Term and Preterm Infants. *Neoreviews*.

[36] Abani O, Abbas A, Abbas F (2021). Convalescent plasma in patients admitted to hospital with COVID-19 (RECOVERY): a randomised controlled, open-label, platform trial. Lancet.

[37] Hammitt LL, Dagan R, Yuan Y (2022). Nirsevimab for Prevention of RSV in Healthy Late-Preterm and Term Infants. *N Engl J Med*.

[38] Organization WH Vaccination schedule for respiratory syncytial virus (RSV). https://immunizationdata.who.int/global/wiise-detail-page/vaccination-schedule-for-respiratory-syncytial-virus-%28rsv%29?ISO_3_CODE=&TARGETPOP_GENERAL=.

[39] Feikin DR, Karron RA, Saha SK (2024). The full value of immunisation against respiratory syncytial virus for infants younger than 1 year: effects beyond prevention of acute respiratory illness. Lancet Infect Dis.

[40] (2025). Tidslinje: myndighetenes håndtering av koronasituasjonen, chronology of government measures in response to the COVID-19 pandemic. https://www.regjeringen.no/no/tema/helse-og-omsorg/folkehelse/tidslinje-koronaviruset/id2692402/.

