# Supplementary material for: Hospitalisations for respiratory tract infections in preterm and term born children aged 1–5 years before, during and after COVID-19 control measures: an interrupted time-series analysis
Source: BMJ Paediatr Open. 2026 Sep 1;10(1):e004270. doi: 10.1136/bmjpo-2025-004270 (PMC13536130; doi:10.1136/bmjpo-2025-004270)
Supplement: Supplementary file 1 [file bmjpo-2025-004270.reviewer_comments.pdf]

PEER REVIEW HISTORY

BMJ Paediatrics Open publishes all reviews undertaken for accepted manuscripts. Reviewers are asked to complete a checklist review form and are provided with free text boxes to elaborate on their assessment. These free text comments are reproduced below.

ARTICLE DETAILS

**TITLE (PROVISIONAL)**	Hospitalizations for respiratory tract infections in preterm and term born children aged 1-5 years before, during, and after COVID-19 control measures: An interrupted time-series analysis
**AUTHORS**	Asheim, Andreas<br>Todd, Isobel<br>Døllner, Henrik<br>Kajantie, Eero<br>Nilsen, Sara Marie<br>Risnes, Kari<br>Opdahl, Signe

VERSION 1 – REVIEW

**REVIEWER NAME**	Foley, Kimberley
**REVIEWER AFFILIATION**	
**REVIEWER CONFLICT OF INTEREST**	No Conflict of Interest
**AI DISCLOSURE**	No, I have not used AI
**DATE REVIEW RETURNED**	12-Dec-2025

**GENERAL COMMENTS**	This is a very interesting and timely paper looking at the impact of COVID-19 control measures on hospitalisations for young children. Importantly, this looks at the impact on children within different gestational groups, which are at higher risk of severe outcomes from RTIs.<br><br>Major comments:<br><br>The introduction/rationale are generally well described and justified. However I would be interested in learn more of the reasons why age 0-<1 were not included in this study? As RSV bronchiolitis infections are very common in this age group, it seems unusual not to include this group and would be very helpful to get a better understanding of why they weren't included and what impact the authors feel this might have on the main messages/interpretation and the implications of this study.<br><br>I would also appreciate a bit of additional detail around the analysis. I understand that the authors used the pre-covid trends to estimate the post-covid rates and then examined the differences in expected vs actual, but I wasn't quite following how this was done. Was the ITS model used to compare the post-covid period to the pre-covid period and what the intervention point (or points) in this model? Were any sensitivity analyses conducted?<br><br>The monthly difference in rates were provided for particular time periods - how was this calculated, was this the mean monthly difference over that time period?

	In the discussion the authors quite rightly point out that the age of exposure to RTIs post-covid may have affected hospitalisations due to differences in immunity. Was the child's age at various time points accounted for in the analysis examining gestational age and hospitalisations?<br><br>The interpretation/implications section was interesting to read, but I think the arguments for improving vaccination rates, and the opportunities around the new RSV immunisation programmes could be further strengthened. (However this also links to my earlier comment above, as I think there's more to consider around the age 0-<1 children that could be mentioned).<br><br>Minor comments:<br><br>There were occasionally very minor grammatical errors (e.g. intro: "Pediatric infection-related hospitalisations affected by these measures, with population studies from..."<br><br>Could the amount of missing data and/or the amount of children excluded due to missing variables be clarified?<br><br>Figure 1 showed the number of admissions and not rates, why was this chosen? For Figure 2, could the x-axis be extended to show the full study period?<br><br>Many thanks for the opportunity to review this paper and I hope these comments are helpful to the authors.

**REVIEWER NAME**	Du Berry, Cassidy
**REVIEWER AFFILIATION**	Murdoch Children's Research Institute
**REVIEWER CONFLICT OF INTEREST**	I have the same affiliations as one of the authors (Isobel Todd); however, I do not work directly with this individual or their research group. I have no other potential competing interests to declare.
**AI DISCLOSURE**	No, I have not used AI
**DATE REVIEW RETURNED**	24-May-2026

**GENERAL COMMENTS**	Thank you for inviting me to review this manuscript. This population-based study uses linked national registry data to evaluate how RTI hospitalisation rates in Norwegian infants and pre-schoolers changed before, during and after the COVID-19 pandemic, and whether these changes differed by gestational status at birth. The authors should be commended on the strength of the dataset (including large sample size), use of nationwide clinical registry data, and relevance of the question in vulnerable population groups. My comments are as follows:<br><br>MAJOR COMMENTS:<br><br>Methods<br><br>Comment #1. Can the authors please state why they chose to group those born 23 to 33 weeks' gestation together, and did not use World Health Organization (and conventionally accepted/used) gestational classifications? (i.e, extremely preterm: <28 weeks' gestation, very preterm: 28 to <32 weeks' gestation, moderate to late preterm: 32 to <37 weeks' gestation).

A 23-week preterm infant/pre-schooler has completely different physiology and risk factors to an age-matched peer born at 33 weeks; from a clinical perspective they are nothing alike (e.g, early-life ventilation, structural lung damage, immunological maturity, and lung function). Pooling them appears to have been done for methodological convenience, a choice perhaps driven by the need to establish an adequate sample size in most gestationally immature preterm group used in this study. This aggregation biases the stratum-level estimate toward the larger and more dominant 32-33 weeks' gestation sub-group, meaning the reported effect for "extremely to moderately preterm" is being driven by the least vulnerable members of that band, undermining the inference the authors are drawing in this manuscript.

A sensitivity analysis should be conducted, splitting preterm groups into extremely preterm (<28wGA), very preterm (28-31wGA), moderate (32-33wGA) and late preterm (34-36wGA). The results of this sensitivity should be compared with the primary analysis.

Comment #2: The exclusion criteria are reasonable but counts of those excluded are not disclosed/reported. Can the authors please include a flow diagram to address this?

Comment #3. It's unclear to me how COVID-19 admissions have been handled. Are RTIs related to COVID-19 included in the outcome? Could the authors clarify this in the methods section? While rare in this age group, it would be useful to know how many children during the study period in Norway were admitted with disease caused by COVID-19 (if the authors have these data).

Comment #4. The ICD-10 codes used in the study are listed but not explained. Could the authors please outline what each code represents and a brief justification for inclusion? This perhaps would be useful as a supplementary table. Doing so would help readers evaluate whether outcome definitions align with the interpretative claims made in the discussion, particularly given the heterogeneity in the codes selected (see comment #6).

Comment #5. Because the at-risk cohort spans children aged 1 to 5 years born between 2012 and 2021, the gestational age composition of the at-risk population turns over month by month (e.g, the 23-33 weeks' GA group in 2018 comprises individuals born 2013 to 2016; the same stratum in late 2022 comprises children born 2017 to 2021). These are different children, born in different obstetric eras, with potentially different exposures from evolving neonatal practices, RSV prophylaxis eligibility, and the pandemic itself. The 2020/2021 birth cohorts are particularly of interest here as they entered the age-1 window during the post-lockdown 'rebound' likely with altered transplacental antibody transfer, reduced infant microbial exposure and (potentially) disrupted vaccination schedules. However, the chosen model treats the population as stationary apart from seasonality, and the analysis cannot separate these different birth year groups. Can the authors please discuss this and acknowledge it as a study limitation?

Discussion

Comment #6. The outcome definition is quite heterogeneous (ICD-codes chosen mix upper RTI and lower RTI, viral and bacterial

	aetiologies, acute and chronic conditions); the authors correctly list this as a study limitation. The discussion section, however, focuses predominantly on lower RTI, particularly RSV. This narrow focus does not align with the broad outcome definition used. Ideally, a sensitivity analysis that narrows the outcome to LRTI-related codes should be performed if the authors which the retain the current framing of the discussion. Alternatively, the authors should consider tempering the RSV-related discussion and speak more to how their findings relate to respiratory illness and admissions more broadly.<br><br>Comment #7. The late preterm group is correctly defined in the manuscript (i.e, 34 to <37 weeks' gestation) but the discussion section as written underreports this gestational stratum despite being the largest preterm subgroup in their dataset. Findings from this study show COVID-19 lockdown measures show a clear impact on this group, but in the discussion, those born late preterm seem to be an afterthought, and this important signal/finding gets buried. In the discussion section, can the authors please expand on what these findings mean for this large gestational group, from both a clinical and policy-level perspective.<br><br>Comment #8. Can the authors please comment in greater detail on specific lockdown measures that were taken by the Norwegian government to combat the spread of SARS-CoV-2? Examples may include commenting on the extent of school closures, national work from home initiatives, social distancing measures, time allowed spent outdoors and distance allowed travelled from home, etc. My (limited) understanding of measures taken was that it was considerably less stringent than other high-income countries such as Australia, Ireland, France and the UK. Further, could the authors please comment on how this may affect the generalisability of their findings?<br><br>MINOR COMMENTS<br><br>Comment #9. Page 5, line 31: Can the authors please explain the concept of "immunity debt".<br><br>Comment #10. Page 18, line 13: a space is missing between "progress" and "planned".

VERSION 1 – AUTHOR RESPONSE

Reviewer: 1
Kimberley Foley
Comments to the Author
This is a very interesting and timely paper looking at the impact of COVID-19 control measures on hospitalisations for young children. Importantly, this looks at the impact on children within different gestational groups, which are at higher risk of severe outcomes from RTIs.

Major comments:

Comment #1
The introduction/rationale are generally well described and justified. However I would be interested in learn more of the reasons why age 0-<1 were not included in this study? As RSV bronchiolitis infections are very common in this age group, it seems unusual not to include this group and would be

very helpful to get a better understanding of why they weren't included and what impact the authors feel this might have on the main messages/interpretation and the implications of this study.

OUR RESPONSE:

The reason was primarily a technical one, since preterm newborns increasingly by lower gestational age at birth require long neonatal hospitalizations and follow up the first year. These are periods of limited exposures to RTIs. Unfortunately, our data were not suitable for appropriately accounting for that, and we therefore chose not to include the first year of life. In the updated manuscript we made sure to include the reasoning behind this choice.

Page 6:

> *"The primary outcome was hospitalisation with a main diagnosis of RTI between ages 1 and 5 years. We excluded hospitalisations during the first year after birth where time at risk was not precise in the data, particularly for preterm groups needing extensive newborn hospital care and follow up. "*

Comment #2
I would also appreciate a bit of additional detail around the analysis. I understand that the authors used the pre-covid trends to estimate the post-covid rates and then examined the differences in expected vs actual, but I wasn't quite following how this was done. Was the ITS model used to compare the post-covid period to the pre-covid period and what the intervention point (or points) in this model? Were any sensitivity analyses conducted?

OUR RESPONSE:

We used a method which is not entirely the typical ITS, since the regular seasonal trends were disrupted during 2020-2021, and pre-specifying the patterns would be problematic. The approach was quite simple: Make a prediction model from the pre-covid rates (jan. 2017- mar. 2020) and predict rates after March 2020. In this sense, March 2020 was the intervention point. The description of the method was minimal in the submitted manuscript, and we realise that a methods supplement may be helpful to better elaborate. We also include additional analyses here to explore the sensitivity of the approach regarding the specification of the prediction step.

Page 7:

> *"Details on the implementation of this method including a sensitivity analysis addressing modelling of seasonality is provided as a methods supplement in the Supplementary Materials, Methods Supplement."*

Comment #3
The monthly difference in rates were provided for particular time periods - how was this calculated, was this the mean monthly difference over that time period?

OUR RESPONSE:

This might have been unclear from a small oversight in the statistics section. We did in fact calculate person time at risk and number of hospitalisations per month, not rates as it said in the submitted version. Rates were calculated subsequently. With time at risk and counts per month, we hope it is now clearer how rates could be calculated in other time-intervals by aggregation.

Page 7, Statistics:

"Monthly number of hospitalisations and person-time at risk were calculated…."

Comment #4
In the discussion the authors quite rightly point out that the age of exposure to RTIs post-covid may have affected hospitalisations due to differences in immunity. Was the child's age at various time points accounted for in the analysis examining gestational age and hospitalisations?

OUR RESPONSE:

The analysis was designed to show differences in rates without any age standardization. We believe the question of differences in the age of exposure and immunity would be of great interest, but that the current analysis is not suited to illuminate this. We also believe that changes in immunity by age are less pronounced after 1 year of age than before. We do however aim to address this in a future work.

Comment #5
The interpretation/implications section was interesting to read, but I think the arguments for improving vaccination rates, and the opportunities around the new RSV immunisation programmes could be further strengthened. (However this also links to my earlier comment above, as I think there's more to consider around the age 0-<1 children that could be mentioned).

OUR RESPONSE:

We have strengthened the interpretation and implications sections by more explicitly emphasising the importance of improving vaccination uptake and by highlighting the opportunities offered by newly introduced RSV immunisation programmes, particularly during the first year of life when preterm-born infants are at highest risk.

Please see updated text on pages 21-21.

Minor comments:

There were occasionally very minor grammatical errors (e.g. intro: "Pediatric infection-related hospitalisations affected by these measures, with population studies from..."

Could the amount of missing data and/or the amount of children excluded due to missing variables be clarified?

Figure 1 showed the number of admissions and not rates, why was this chosen? For Figure 2, could the x-axis be extended to show the full study period?

Many thanks for the opportunity to review this paper and I hope these comments are helpful to the authors.

OUR RESPONSE:

We have done a thorough read-through and caught some errors, including the one you point to.

In the updated version we provide a flow-chart showing the data selection, hopefully making the issue of missing variables clearer.

We choose to show numbers in Figure 1 to highlight how the healthcare services were affected, rather than particular groups.

We considered extending the x-axis in Figure 2, but this may make the figure hard to read. In the updated appendix, under the sensitivity analysis, we include a figure which has the whole axis. We hope this may address any concerns.

Thank you for taking the time to read out manuscript and provide us some very helpful feed-back.

Reviewer: 2
Dr. Cassidy Du Berry, Murdoch Children's Research Institute

Comments to the Author
Thank you for inviting me to review this manuscript. This population-based study uses linked national registry data to evaluate how RTI hospitalisation rates in Norwegian infants and pre-schoolers changed before, during and after the COVID-19 pandemic, and whether these changes differed by gestational status at birth. The authors should be commended on the strength of the dataset (including large sample size), use of nationwide clinical registry data, and relevance of the question in vulnerable population groups. My comments are as follows:

MAJOR COMMENTS:

Methods

Comment #1

Can the authors please state why they chose to group those born 23 to 33 weeks' gestation together, and did not use World Health Organization (and conventionally accepted/used) gestational classifications? (i.e, extremely preterm: <28 weeks' gestation, very preterm: 28 to <32 weeks' gestation, moderate to late preterm: 32 to <37 weeks' gestation).

A 23-week preterm infant/pre-schooler has completely different physiology and risk factors to an age-matched peer born at 33 weeks; from a clinical perspective they are nothing alike (e.g, early-life ventilation, structural lung damage, immunological maturity, and lung function). Pooling them appears to have been done for methodological convenience, a choice perhaps driven by the need to establish an adequate sample size in most gestationally immature preterm group used in this study. This aggregation biases the stratum-level estimate toward the larger and more dominant 32-33 weeks' gestation sub-group, meaning the reported effect for "extremely to moderately preterm" is being driven by the least vulnerable members of that band, undermining the inference the authors are drawing in this manuscript. A sensitivity analysis should be conducted, splitting preterm groups into extremely preterm (<28wGA), very preterm (28-31wGA), moderate (32-33wGA) and late preterm (34-36wGA). The results of this sensitivity should be compared with the primary analysis.

OUR RESPONSE:

The extremely, very and moderately preterm groups are quite small, so this grouping was done to achieve some statistical power.

We see that this choice is a valid reason for concern, among the extremely to very preterm born rates for the most vulnerable will likely be drowned out. We have done an additional analysis showing the rates for the WHO GA categories separated to assess how this might impact the results. This is now included as Supplementary Figure 2.

Page 6:

> *"The grouping of extreme (<28 weeks), very (28-31), and moderately (32-33) preterm into one group was presented as main analysis, while rates for each WHO category were presented in supplementary analysis.*

Results section updated (p 10):

> *"Considering the extremely, very and moderately preterm born separately in an additional analysis (Supplementary Figure 2) indicates that rates were substantially higher by lower gestational age and highest for the extremely preterm born."*

Comment #2

The exclusion criteria are reasonable but counts of those excluded are not disclosed/reported. Can the authors please include a flow diagram to address this?

OUR RESPONSE:

We have now included a flow-chart in the Supplementary Materials. When making this, we also uncovered some errors in the descriptive table, which has been updated. The analyses were not affected by this error.

Page 6:

> *"A flowchart including the number of excluded at each stage is provided in Supplementary Figure 1."*

Comment #3

It's unclear to me how COVID-19 admissions have been handled. Are RTIs related to COVID-19 included in the outcome? Could the authors clarify this in the methods section? While rare in this age group, it would be useful to know how many children during the study period in Norway were admitted with disease caused by COVID-19 (if the authors have these data).

OUR RESPONSE:

The ICD-10 codes used to specify RTIs include specific Covid codes and unspecified acute LRTI infections (and J12.2 and J22). These could have been used whenever there was a Covid infection. In addition, two special purpose codes were introduced to specify: U07.1, COVID-19, virus identified. U07.2, COVID-19, virus not identified.

As the results were presented, Covid admissions were therefore included. In the updated manuscript we made sure this was clear. We have also included the special purpose code in the code description, and we also included the number of likely Covid admissions in the Results section for context.

Page 7:

> *« In total, we identified 6983 hospitalisations with RTIs from 2017 to 2022 among 265 055 children. After March 2020 there were 4535 RTI hospitalisations, among which 444 were indicated as likely COVID-19 related.»*

Comment #4

The ICD-10 codes used in the study are listed but not explained. Could the authors please outline what each code represents and a brief justification for inclusion? This perhaps would be useful as a supplementary table. Doing so would help readers evaluate whether outcome definitions align with the interpretative claims made in the discussion, particularly given the heterogeneity in the codes selected (see comment #6).

OUR RESPONSE:

We have added some explanation to make this clearer.

Page 6:

> *“we considered all the following codes as indication of RTI: J00-J06, J09-J18, J20-22, J32, J39.0, J39.1, J85, and J86. These include bacterial and viral upper and lower respiratory tract infections. “*

We have also included a list of the ICD-10 codes in the updated Supplementary materials with more details (Supplementary table 1).

Comment #5

Because the at-risk cohort spans children aged 1 to 5 years born between 2012 and 2021, the gestational age composition of the at-risk population turns over month by month (e.g, the 23-33 weeks’ GA group in 2018 comprises individuals born 2013 to 2016; the same stratum in late 2022 comprises children born 2017 to 2021). These are different children, born in different obstetric eras, with potentially different exposures from evolving neonatal practices, RSV prophylaxis eligibility, and the pandemic itself. The 2020/2021 birth cohorts are particularly of interest here as they entered the age-1 window during the post-lockdown ‘rebound’ likely with altered transplacental antibody transfer, reduced infant microbial exposure and (potentially) disrupted vaccination schedules. However, the chosen model treats the population as stationary apart from seasonality, and the analysis cannot separate these different birth year groups. Can the authors please discuss this and acknowledge it as a study limitation?

OUR RESPONSE:

We have added in limitations, page 17:

> *“The analysis included individuals born during a time span of 10 years, when the distribution of the youngest gestational age group may have changed marginally due to increased survival. Also, in 2021, during re-surge, the children in 1-5 year group would have different immunity since the youngest were born during the pandemic with altered transplacental antibody transfer, reduced infant microbial exposure and (potentially) disrupted vaccination schedules. When using a repeated cross sectional design, results could have been affected by such differences between birth cohorts and their histories at different times of the study.”*

Discussion

Comment #6

The outcome definition is quite heterogeneous (ICD-codes chosen mix upper RTI and lower RTI, viral and bacterial aetiologies, acute and chronic conditions); the authors correctly list this as a study limitation. The discussion section, however, focuses predominantly on lower RTI, particularly RSV. This narrow focus does not align with the broad outcome definition used. Ideally, a sensitivity analysis that narrows the outcome to LRTI-related codes should be performed if the authors which the retain

the current framing of the discussion. Alternatively, the authors should consider tempering the RSV-related discussion and speak more to how their findings relate to respiratory illness and admissions more broadly.

OUR RESPONSE:

Hospitalizations for RTI in children 1-5 years are dominated by LRTIs and within LRTIs viruses, mainly RSV is predominant. In the updated manuscript, we have done an additional analysis on LRTIs, showing similar results and we have added references to underpin this. Since RSV is likely to be dominating other pathogens in this context, and we have in made sure to elaborate this point.

Page 14:

> "Among the RTI hospitalisations in our study, RSV was likely a major contributor,[16] and the marked and rapid reduction in childhood RTI and LRTI hospitalisation observed during the COVID-19 pandemic is therefore in line with these studies."

References 16 has been added in update:

16. Hasegawa K, Mansbach JM, Teach SJ, et al. Multicenter study of viral etiology and relapse in hospitalized children with bronchiolitis. The Pediatric infectious disease journal. 2014;33(8):809–813.

Comment #7

The late preterm group is correctly defined in the manuscript (i.e, 34 to <37 weeks' gestation) but the discussion section as written underreports this gestational stratum despite being the largest preterm subgroup in their dataset. Findings from this study show COVID-19 lockdown measures show a clear impact on this group, but in the discussion, those born late preterm seem to be an afterthought, and this important signal/finding gets buried. In the discussion section, can the authors please expand on what these findings mean for this large gestational group, from both a clinical and policy-level perspective.

OUR RESPONSE:

We have revised the Discussion to more explicitly address children born late preterm, highlighting their numerical dominance among preterm births and the population-level relevance of the observed changes in hospitalisation. We also expand on the clinical and policy implications of these findings, particularly in relation to infection prevention and evolving RSV immunisation strategies.

See second paragraph of the interpretation, pages 18-19.

> *"Although the highest absolute hospitalisation rates were observed among the rather wide gestational age range of children born extremely to moderately preterm, an additional analysis shows that the extremely preterm born were particularly vulnerable. Nevertheless, the preterm population is numerically dominated by children born late preterm. Even modest relative changes in hospitalisation risk in this group therefore translate into substantial absolute numbers s at the population level. The marked reduction in RTI hospitalisation among late preterm children during periods of COVID-19 measures, followed by a clear rebound after measures were lifted, highlights that this large gestational subgroup is highly responsive to changes in community transmission. From a policy perspective, these findings are relevant as children born late preterm are in many cases not included in targeted prevention strategies, including vaccination policies and RSV prevention programmes. The observed sensitivity of this group to changes in infection pressure suggest that preventive measures with broader*

> *reach among preterm groups, such as vaccination, RSV immunisation programmes and infection prevention strategies, may have substantial public health impact beyond targeting only the most extremely preterm infants. "*

Comment #8

Can the authors please comment in greater detail on specific lockdown measures that were taken by the Norwegian government to combat the spread of SARS-CoV-2? Examples may include commenting on the extent of school closures, national work from home initiatives, social distancing measures, time allowed spent outdoors and distance allowed travelled from home, etc. My (limited) understanding of measures taken was that it was considerably less stringent than other high-income countries such as Australia, Ireland, France and the UK. Further, could the authors please comment on how this may affect the generalisability of their findings?

OUR RESPONSE:

In the updated manuscript, we have addressed how Norway's handling of the pandemic may limit generalisability.

Third paragraph of Strengths and limitations, page 17:

> *"Norway implemented a range of non-pharmaceutical interventions to limit the spread of COVID-19 during 2020-2021, including periods of school and kindergarten closures, work-from-home recommendations, restrictions on social gatherings, reduced travel, and general guidance on physical distancing, while access to outdoor activities was largely maintained.[30,31] Stringency and duration of measures varied substantially across European countries.[32] The relatively shorter and less continuous restrictions in Norway, together with cross-country differences in compliance and context, may limit generalisability of our findings. "*

MINOR COMMENTS

Comment #9. Page 5, line 31: Can the authors please explain the concept of "immunity debt".

Comment #10. Page 18, line 13: a space is missing between "progress" and "planned".

OUR RESPONSE:

These comments have been addressed in the updated manuscript.

Thank you for taking the time to provide us some very insightful feed-back on our work.

Reviewer: 1
If you have selected 'Yes' above, please provide details of any competing interests.: Not applicable

Reviewer: 2
If you have selected 'Yes' above, please provide details of any competing interests.: I have the same affiliations as one of the authors (Isobel Todd); however, I do not work directly with this individual or their research group. I have no other potential competing interests to declare.

VERSION 2 – REVIEW

**REVIEWER NAME**	Foley, Kimberley
**REVIEWER AFFILIATION**	
**REVIEWER CONFLICT OF INTEREST**	I am a co-investigator on a study examining the impact of the maternal RSV vaccine on healthcare activity in Northwest London. This funding for this investigator-led study was provided by Sanofi & AstraZeneca and all funds were paid to Imperial College.
**AI DISCLOSURE**	No, I have not used AI
**DATE REVIEW RETURNED**	06-Aug-2026

**GENERAL COMMENTS**	Thank you for your response and careful attention to my comments and questions. I have no additional comments.

**REVIEWER NAME**	Du Berry, Cassidy
**REVIEWER AFFILIATION**	Murdoch Children's Research Institute
**REVIEWER CONFLICT OF INTEREST**	I have the same affiliations as one of the authors (Isobel Todd); however, I do not work directly with this individual or their research group. I have no other potential competing interests to declare.
**AI DISCLOSURE**	No, I have not used AI
**DATE REVIEW RETURNED**	04-Aug-2026

**GENERAL COMMENTS**	Thank you for addressing my comments. I have no further suggestions. The manuscript is of high quality, and I recommend acceptance.
