# Supplementary material for: Hospitalisations for respiratory tract infections in preterm and term born children aged 1–5 years before, during and after COVID-19 control measures: an interrupted time-series analysis
Source: BMJ Paediatr Open. 2026 Sep 1;10(1):e004270. doi: 10.1136/bmjpo-2025-004270 (PMC13536130; doi:10.1136/bmjpo-2025-004270)
Supplement: Supplementary file 2 [file bmjpo-10-1-s001.docx]

**Supplementary figure 1:** Flow-chart of births included in the study

**Supplementary table 1:**  List of ICD-10 codes used to identify hospitalisations for respiratory tract infections with grouping.

| Acute upper respiratory infections | | |
| --- | --- | --- |
|  | **J00** | Acute nasopharyngitis (common cold) |
|  | **J01** | Acute sinusitis |
|  | **J02** | Acute pharyngitis |
|  | **J03** | Acute tonsillitis |
|  | **J04** | Acute laryngitis and tracheitis |
|  | **J05** | Acute obstructive laryngitis (croup) and epiglottitis |
|  | **J06** | Acute upper respiratory infections of multiple and unspecified sites |
| Influenza and pneumonia | | |
|  | **J09** | Influenza due to certain identified influenza viruses |
|  | **J10** | Influenza due to other identified influenza virus |
|  | **J11** | Influenza, virus not identified |
|  | **J12** | Viral pneumonia, not elsewhere classified |
|  | **J13** | Pneumonia due to Streptococcus pneumoniae |
|  | **J14** | Pneumonia due to Haemophilus influenzae |
|  | **J15** | Bacterial pneumonia, not elsewhere classified |
|  | **J16** | Pneumonia due to other infectious organisms, not elsewhere classified |
|  | **J17** | Pneumonia in diseases classified elsewhere |
|  | **J18** | Pneumonia, organism unspecified |
| Other acute lower respiratory infections | | |
|  | **J20** | Acute bronchitis |
|  | **J21** | Acute bronchiolitis |
|  | **J22** | Unspecified acute lower respiratory infection |
| Chronic sinusitis | | |
|  | **J32** | Chronic sinusitis |
| Other diseases of upper respiratory tract | | |
|  | **J39.0** | Retropharyngeal and parapharyngeal abscess |
|  | **J39.1** | Other abscess of pharynx |
| Suppurative and necrotic conditions of lower respiratory tract | | |
|  | **J85** | Abscess of lung and mediastinum |
|  | **J86** | Pyothorax |

In addition, we used the following special purpose codes to indicate likely COVID-19 admissions:

|  | **U07.1** | COVID‑19, virus identified |
| --- | --- | --- |
|  | **U07.2** | COVID-19, virus not identified |

**Methods supplement**

*Prediction algorithm*

The analyses of this work were implemented as an Interrupted Time Series analysis, in the sense that a pre-intervention, i.e., pre-covid, pattern of hospitalisations for respiratory tract infections were compared with actual hospitalisations post intervention. We made no assumptions regarding seasonality in the post intervention phase; the results were based on monthly comparison between rates of admission predicted from data pre covid and actual rates of hospitalisations from March 2020.

The prediction model we used was a Poisson-type model, where an offset parameter accounted for the number of individuals under risk during each month,

$\log\left( E\left[ \frac{Admissions(t)}{time at risk(t)} \right] \right)={\beta_{1}^{s}s}_{1}\left( t \right)+{\beta_{1}^{c}c}_{1}\left( t \right)+{\beta_{2}^{s}s}_{2}\left( t \right)+{\beta_{1}^{c}c}_{2}\left( t \right)-log(time at risk(t))$.

The predictors were harmonic terms that modelled a harmonic recurring pattern throughout a year,

$$s_{i}\left( t \right)=\sin\left( 2\pi\frac{i\cdot t}{12} \right), c_{i}\left( t \right)=cos\left( 2\pi\frac{i\cdot t}{12} \right).$$

The model was fitted with a quasi-Poisson method, *glm(family=quasipoisson)* in R.

*Uncertainty quantification*

Precision was evaluated with bootstrapping by resampling the monthly rates 10 000 times. For each resample, a predicted expected value per month was computed which was then used to draw a realisation from a Poisson-distribution.

*Sensitivity analyses*

To assess the sensitivity of the analyses to the prediction, we ran the main analysis with two variations in how seasonality was modelled.

In the sensitivity analyses, we varied the level of detail in the modelling of seasonality and investigated how this affected results. We first included only the basic harmonic, meaning we removed $s_{2}\left( t \right)$ and $c_{2}\left( t \right)$. Secondly, we included the third harmonic, meaning we added $s_{3}\left( t \right)$ and $c_{3}\left( t \right)$. The results of these analyses are included in the below figure, which show negligible difference between the three specifications.

**Supplementary table 2:** Number of RTI hospital admissions by month and year, among children aged 1-5 years, born in Norway 2012-2021.

*The pandemic period is highlighted in grey.*

|  | 2017 | 2018 | 2019 | 2020 | 2021 | 2022 |
| --- | --- | --- | --- | --- | --- | --- |
| Jan | 285 | 232 | 267 | 192 | 55 | 118 |
| Feb | 222 | 230 | 258 | 212 | 49 | 110 |
| Mar | 142 | 202 | 175 | 133 | 66 | 148 |
| Apr | 106 | 139 | 108 | 24 | 55 | 154 |
| May | 135 | 113 | 101 | 22 | 54 | 150 |
| Jun | 105 | 108 | 72 | 48 | 73 | 137 |
| Jul | 62 | 61 | 51 | 40 | 34 | 75 |
| Aug | 66 | 75 | 71 | 55 | 62 | 121 |
| Sep | 132 | 117 | 113 | 73 | 210 | 216 |
| Oct | 161 | 120 | 128 | 70 | 367 | 203 |
| Nov | 196 | 140 | 147 | 88 | 348 | 217 |
| Dec | 188 | 206 | 173 | 73 | 188 | 287 |

*RTI – respiratory tract infection*

**Supplementary table 3:** Rates of RTI hospitalization and observed person time during and after the pandemic, among children aged 1-5 years, born in Norway 2012-2021.

*Rates of admissions per 1000 person-years*

|  | extremely to moderately preterm  (23–33 weeks) | |  | late preterm  (34–36 weeks) | |  | early to full-term  (37–41 weeks) | |
| --- | --- | --- | --- | --- | --- | --- | --- | --- |
|  | rate | 1000 p.y. |  | rate | 1000 p.y. |  | rate | 1000 p.y. |
| May 2020 | 0 | 0.23 |  | 2 | 0.51 |  | 1 | 14.84 |
| June 2020 | 17 | 0.23 |  | 4 | 0.51 |  | 3 | 14.84 |
| July 2020 | 13 | 0.23 |  | 4 | 0.51 |  | 2 | 14.80 |
| August 2020 | 22 | 0.23 |  | 8 | 0.50 |  | 3 | 14.75 |
| September 2020 | 61 | 0.23 |  | 4 | 0.50 |  | 4 | 14.70 |
| October 2020 | 13 | 0.23 |  | 10 | 0.50 |  | 4 | 14.63 |
| November 2020 | 30 | 0.23 |  | 10 | 0.50 |  | 5 | 14.57 |
| December 2020 | 22 | 0.23 |  | 2 | 0.51 |  | 5 | 14.59 |
| January 2021 | 17 | 0.23 |  | 6 | 0.50 |  | 3 | 14.60 |
| February 2021 | 13 | 0.23 |  | 6 | 0.50 |  | 3 | 14.57 |
| March 2021 | 13 | 0.23 |  | 4 | 0.50 |  | 4 | 14.56 |
| April 2021 | 9 | 0.23 |  | 8 | 0.50 |  | 3 | 14.54 |
| May 2021 | 26 | 0.23 |  | 8 | 0.50 |  | 3 | 14.51 |
| June 2021 | 18 | 0.23 |  | 6 | 0.50 |  | 5 | 14.48 |
| July 2021 | 9 | 0.23 |  | 6 | 0.49 |  | 2 | 14.46 |
| August 2021 | 27 | 0.22 |  | 18 | 0.49 |  | 3 | 14.40 |
| September 2021 | 63 | 0.22 |  | 22 | 0.49 |  | 13 | 14.35 |
| October 2021 | 113 | 0.22 |  | 62 | 0.49 |  | 22 | 14.29 |
| November 2021 | 100 | 0.22 |  | 52 | 0.49 |  | 21 | 14.24 |
| December 2021 | 41 | 0.22 |  | 25 | 0.49 |  | 12 | 14.25 |
| January 2022 | 36 | 0.22 |  | 12 | 0.48 |  | 7 | 14.27 |
| February 2022 | 14 | 0.22 |  | 16 | 0.49 |  | 7 | 14.29 |
| March 2022 | 45 | 0.22 |  | 23 | 0.49 |  | 9 | 14.33 |
| April 2022 | 55 | 0.22 |  | 27 | 0.49 |  | 9 | 14.36 |
| May 2022 | 60 | 0.22 |  | 12 | 0.49 |  | 9 | 14.38 |
| June 2022 | 37 | 0.22 |  | 26 | 0.49 |  | 8 | 14.40 |
| July 2022 | 9 | 0.22 |  | 6 | 0.49 |  | 5 | 14.42 |
| August 2022 | 32 | 0.22 |  | 8 | 0.49 |  | 8 | 14.41 |
| September 2022 | 69 | 0.22 |  | 21 | 0.49 |  | 13 | 14.40 |
| October 2022 | 60 | 0.22 |  | 25 | 0.48 |  | 12 | 14.38 |
| November 2022 | 83 | 0.22 |  | 29 | 0.48 |  | 13 | 14.34 |
| December 2022 | 76 | 0.21 |  | 46 | 0.47 |  | 18 | 14.09 |
| *p.y. - person-years, RTI – respiratory tract infection* | | | | | | | | |

**Supplementary Figure 2**: Rates of hospitalisations due to respiratory tract infections during and after the pandemic among children who were extremely to moderately preterm born (blue dots), aged 1-5 years. Predicted rates of admission based on pre-pandemic admissions, calculated with quasi-Poisson regression with harmonic seasonal terms, is shown as a continuous curve with a 95% confidence band. Each panel shows, in addition, rates of admissions for extremely, very and moderately preterm born respectively.

**Supplementary table 4:** Observed versus predicted rates of LRTI admissions, per pandemic year, among children aged 1-5 years born in Norway 2012-2021, according to gestational age at birth.

|  |  | extremely to moderately preterm  (23–33 weeks) | late preterm  (34–36 weeks) | early to full-term  (37–41 weeks) |
| --- | --- | --- | --- | --- |
| Apr 2020 to March 2021 | |  |  |  |
|  | Rate per 1000 person-years | 9 | 1·9 | 0·8 |
|  | Difference vs predicted rates | -31 (-46 to -19) | -9.2 (-16 to -4·7) | -5.0 (-6·7 to -3·8) |
| Apr 2021 to March 2022 | |  |  |  |
|  | Rate per 1000 person-years | 26 | 10 | 4·4 |
|  | Difference vs predicted rates | 13 (-28 to -2) | -0·1 (-6·6 to 3·9) | -1·1 (-2·8 to 0·1) |
| Apr 2022 to Dec 2022 | |  |  |  |
|  | Rate per 1000 person-years | 22 | 7.1 | 2·5 |
|  | Difference vs predicted rates | 1.1 (-11 to 10) | 1·3 (-4·7 to 5.4) | -0·8 (-2·2 to 0·1) |

*Predicted rates of admission based on pre-pandemic admissions, calculated with quasi-Poisson regression with harmonic seasonal terms. Bootstrapped 95% confidence intervals.*

**Supplementary Table 5**: Monthly cumulative difference between the predicted and actual number of RTI admissions, among children aged 1-5 years, born in Norway 2012-2021.

*The predicted number of admissions were based on pre-pandemic admissions (January 2017 – March 2020), calculated with quasi-Poisson regression with harmonic seasonal terms. Monthly estimates presented with a 95% bootstrapped confidence interval.*

|  | Difference between observed and predicted numbers of RTI hospitalisations (95% CI) | | |
| --- | --- | --- | --- |
|  | extremely to moderately preterm  (23–33 weeks) | late preterm  (34–36 weeks) | early to full-term  (37–41 weeks) |
| March 2020 | -14 (-45, 15) | 5 (-24, 33) | -93 (-242, 54) |
| June 2020 | -29 (-63, 3) | -9 (-41, 20) | -272 (-440, -110) |
| September 2020 | -23 (-59, 10) | -18 (-53, 14) | -354 (-533, -178) |
| December 2020 | -37 (-77, -1) | -35 (-73, -1) | -582 (-791, -384) |
| March 2021 | -72 (-116, -31) | -64 (-106, -24) | -953 (-1187, -723) |
| June 2021 | -80 (-127, -35) | -70 (-114, -29) | -1053 (-1305, -805) |
| September 2021 | -74 (-123, -27) | -64 (-111, -19) | -1006 (-1272, -746) |
| December 2021 | -46 (-98, 3) | -24 (-73, 23) | -651 (-938, -371) |
| March 2022 | -68 (-125, -15) | -34 (-87, 17) | -835 (-1150, -522) |
| June 2022 | -54 (-113, 3) | -19 (-75, 34) | -715 (-1048, -384) |
| September 2022 | -46 (-107, 13) | -18 (-78, 38) | -559 (-902, -220) |
| December 2022 | -26 (-90, 36) | 3 (-59, 61) | -370 (-742, -7) |
